# Martial Art Training and Cognitive Performance in Middle-Aged Adults

**DOI:** 10.1515/hukin-2015-0083

**Published:** 2015-10-14

**Authors:** Peter Douris, Christopher Douris, Nicole Balder, Michael LaCasse, Amir Rand, Freya Tarapore, Aleskey Zhuchkan, John Handrakis

**Affiliations:** 1 Department of Physical Therapy, New York Institute of Technology, Old Westbury, NY.

**Keywords:** executive function, exercise, aging, stroop, martial arts

## Abstract

Cognitive performance includes the processes of attention, memory, processing speed, and executive functioning, which typically declines with aging. Previous research has demonstrated that aerobic and resistance exercise improves cognitive performance immediately following exercise. However, there is limited research examining the effect that a cognitively complex exercise such as martial art training has on these cognitive processes. Our study compared the acute effects of 2 types of martial art training to aerobic exercise on cognitive performance in middle-aged adults. We utilized a repeated measures design with the order of the 3 exercise conditions randomly assigned and counterbalanced. Ten recreational middle-aged martial artists (mean age = 53.5 ± 8.6 years) participated in 3 treatment conditions: a typical martial art class, an atypical martial art class, and a one-hour walk at a self-selected speed. Cognitive performance was assessed by the Stroop Color and Word test. While all 3 exercise conditions improved attention and processing speed, only the 2 martial art conditions improved the highest order of cognitive performance, executive function. The effect of the 2 martial art conditions on executive function was not different. The improvement in executive function may be due to the increased cortical demand required by the more complex, coordinated motor tasks of martial art exercise compared to the more repetitive actions of walking.

## Introduction

Executive function is the highest order of cognitive function involved in selective attention, judgment, anticipation, planning, and conflict resolution through selective inhibition occurring in the prefrontal cortex (Tam, 2013). Research supports the beneficial acute effects of moderate aerobic exercise on cognitive performance for persons of all ages (Barella et al., 2010; Netz et al., 2007; Netz et al., 2009). Netz et al. (2007) concluded that a single bout of moderate aerobic exercise was sufficient for an acute improvement in cognitive processes in late middle-aged adults. Alves et al. (2012) demonstrated that acute bouts of strength and aerobic exercise improved cognitive performance equally as measured by the Stroop test in middle-aged women. The effects of quantity (intensity and duration) of exercise on cognitive performance have been well documented; however, the effect of qualitative mode of exercise as a mediator of cognitive performance remains under-investigated, according to Pesce (2012). There is limited research supporting martial arts as a mediator or enhancer of cognitive function. Martial art training may be considered a mediator of cognitive function as it is a mind-body exercise requiring greater cortical recruitment due the inherently more complex movement patterns compared to the more repetitive actions of the aerobic or resistance exercises that have already been investigated. Chang et al. (2010) stated that Tai Chi was shown to provide aerobic and muscular fitness while imposing greater cognitive involvement, i.e., concentration, focus, imagery and mindfulness that was not required in a typical aerobic or weight training session. Yoga, when performed in conjunction with Tai Chi has been shown to improve the speed and accuracy of mathematical computations in adults (Field et al., 2010). Executive function was enhanced following an acute bout of yoga, however, was not improved by aerobic exercise in college-aged women (Gothe et al., 2013). Winstein et al. (1997) discovered that the cerebral blood flow to areas responsible for cognition rose as the complexities of the movement patterns increased.

The majority of research has focused on the acute effects of aerobic and strengthening exercises on cognitive performance. However, recently alternate forms of exercise have also been investigated (Field et al., 2010; Gothe et al., 2013). In the current study, we planned to address this gap in the knowledge by measuring the acute effects of martial art exercise on cognitive performance in middle-aged adults. Our primary hypothesis was that martial art training would have a greater positive effect on cognitive performance in middle-aged adults than walking because of the increased cortical demand necessary for the complex motor tasks of martial arts compared to the more repetitive actions of walking. Our secondary hypothesis was that an atypical martial art class which consisted of experienced martial artists performing typical training movements in an unfamiliar manner, would have a greater effect on cognitive performance than the well-rehearsed movements of a typical martial art class due to the increased cortical demand of the former.

## Material and Methods

### Participants

Ten participants who were enrolled in a martial arts school of Soo Bahk Do (SBD), a traditional Korean martial art, 4 women and 6 men (mean age = 53.5 ± 8.6 years) volunteered to participate in this study. The subjects were selected according to the inclusion/exclusion criteria. Inclusion criteria were as follows: age range: 40–65 years, good overall health, had attained the rank of black belt, regularly attended one-hour martial arts classes at least two days/week, normal corrected vision and color vision. The following conditions were considered as exclusion criteria: any major musculoskeletal injuries over the last 6 months and any other health issues that would interfere with subject’s safety during exercise. Volunteers completed a medical history and health questionnaire in order to screen for the inclusion and exclusion criteria. Descriptive information of the participants is presented in Table 1. The subjects first reported to the martial art school where they received a detailed explanation of the study’s procedures, risks, benefits and possible discomforts. Afterwards, they gave their signed consent in order to participate in the study. All procedures of the investigation were conducted in accordance with the Helsinki Declaration of 1975. The consent form and the study were approved by the Institutional Review Board of the New York Institute of Technology.

### Measures

Cognitive performance was assessed by the Stroop test. The Stroop test is a timed performance task that consists of three sections: Word section composed of names of three colors in black font, Color section of rows of the letter “X” with each “X” in one of the three different colors, and Color–Word section that consists of words from the first page but with the font of each word in a color that conflicts with the color that the word described. Subjects were instructed to read the words or name the font colors as quickly as possible for 45 s for the Word and Color. The third part of the test presents the two conflicting sources of color information causing a competing effect known as Stroop interference. A conflict arises between reflexively reading the word’s description or naming the color of the font. The individual must choose one response by suppressing the other choice (response inhibition). The Stroop test is frequently utilized to assess the effects of acute exercise on cognitive performance (Tam, 2013; Barella et al., 2010; Alves et al., 2014). The Word and Color T-scores assess attention and processing speed, while the Color-Word and Interference T-scores assess response inhibition which is an index of executive function. Higher scores represent superior cognitive performance (Barella et al., 2010).

### Procedures

The research design utilized was repeated measures and this design is one of the most efficient methods for controlling for intersubject differences as subjects act as their own control (Portney and Watkins, 2009). Participants took part in three separate one–hour exercise conditions: 1) a typical or traditional martial art class, 2) an atypical martial art class, 3) a one-hour brisk walk at a self-selected pace. The walk was chosen as a control condition as our aim was to differentiate the effects of martial art versus repetitive aerobic exercise and walking at a moderate pace had already been shown to improve cognitive performance (Barella et al., 2010; Netz et al., 2007; Netz et al., 2009). We opted to utilize recreational martial artists taking into consideration that they already possessed the specific skill set necessary to perform the traditional martial art movements as well as the walking condition. The middle-aged population was chosen as this is the age when cognitive performance usually begins to decline (Bixby et al., 2007; Cohn et al., 1984; Elst et al., 2006).

The study took place between 7:30 and 9:30 AM on a Saturday for each condition. The treatment conditions were separated by one week to reduce practice effects. The order of exercise conditions was randomly assigned and counterbalanced according to a Latin square for three conditions, in order to also ensure the practice effects of the Stroop test were equally distributed across all the conditions of the experiment (Portney and Watkins, 2009). The independent variables were the three exercise conditions and the dependent variable was the Stroop test which measures the cognitive performance processes of attention, processing speed, and executive function. A certified master instructor in SBD was the instructor for each martial art class. A typical SBD class is one hour in length. Our typical class consisted of a 5 min warm up of flexibility exercises, 10 min of blocking and punching drills, 10 min of kicking, 10 min of hyung (set forms), 15 min of self-defense techniques, and 5 min of sparring, ending with a 5 min cool down (Douris et al., 2004). The atypical class involved the same time frame and order of practice techniques except participants practiced their accustomed techniques to the opposite side, at various speeds, as well as in reverse of the typical movement pattern, practicing their normal techniques in an unaccustomed manner. For the walk exercise condition, the subjects were instructed to walk at a self-selected moderate pace outdoors for one hour. Prior to each exercise condition, participants were administered the Stroop test to assess their attention, processing speed, and executive function. Immediately after exercise, participants were administered a second Stroop test.

### Statistical analyses

Statistical analyses were performed utilizing SPSS for Windows (version 22.0, Chicago, Ill.), using a multifactor repeated measures design. In order to test the hypothesis, we performed a 2 × 3 repeated measures ANOVA for each individual Stroop T-score, with time (pre and post exercise) and exercise (3 conditions) as the main effects. The assumption of sphericity was tested using the Mauchly’s test, in the event that sphericity was violated a Greenhouse-Geisser correction factor was applied. In order to test the main hypothesis that martial art training can improve cognitive performance, a priori pairwise comparisons of the simple effects were conducted when there was a significant main effect for time (pre to post) without a significant interaction effect. According to Tybout et al. (2001), it is appropriate to test for simple effects when an a priori expectation exists even though the interaction term fails to achieve significance. The magnitudes of the difference between significant pairwise comparisons were calculated by the effect size utilizing Cohen’s *d*. The magnitude of the difference was considered as small (0.2 to 0.5), medium (0.5 to 0.8), and large (>0.8) (Portney and Watkins, 2009). A priori sample size calculation revealed that 10 subjects were required in each group in order to detect observed differences at a power of 80%. The level of significance was set at p<0.05.

## Results

The means and standard deviations of the four Stroop T-scores, pre and post exercise are presented in Table 2. In the Word test, the main effect for condition was not significant (*F* (2, 18) =.505, *p*=0.54). The interaction of time x condition was also not significant (*F (*2, 18) =.774, *p*=0.48). A significant main effect was found for time (*F* (1, 9) =123.84, *p*=0.001). Pairwise comparisons with paired t-tests for comparing the pre to post scores revealed there was a significant difference for the typical class (*p*=0.001, *d*=1.8), the walk (*p*=0.001, *d*= 1.6) and for the atypical class (*p*=0.005, *d*= 1.1). These results demonstrate that all three conditions were effective in improving the word task.

In the Color test, the main effect for condition was not significant (*F (*2, 18) =.193, *p*=0.71). The interaction of time x condition was also not significant (*F (*2, 18) =.487, *p*=0.62). A significant main effect was found for time (*F* (1, 9) =28.968, *p*=0.001). Pairwise comparisons with paired t-tests comparing pre to post scores revealed there was a significant difference for the typical class (*p*=0.001, *d*=1.6), the walk (*p*=0.001, *d*=1.6) and for the atypical class (*p*=0.02, *d*=0.8). These results demonstrate that all three conditions were effective in improving performance during the Color task.

In the Color-Word test, the main effect for condition was not significant (*F (*2, 18)=.234, *p*=0.79). The interaction of time x condition was also not significant (*F (*2, 18)=1.644, *p*=0.22). A significant main effect was found for time, (*F* (1, 9)=17.056, *p*=0.003). Pairwise comparisons with paired t-tests comparing pre to post scores revealed there was a significant difference for the typical class (*p*=0.002, *d*=1.4), the atypical class (*p*=.03, *d*=0.7), but not for the walk condition (*p*=0.09). These results demonstrate that only the martial art classes were effective in improving the Color-Word task.

For the interference scores, the main effect for condition was not significant (*F (*2, 18) =.282, *p*=0.76). The interaction of time x condition was also not significant (*F (*2, 18) =1.841, *p*=0.19). A significant main effect was found for time (*F* (1, 9) =8.824, *p*=0.02). Pairwise comparisons with paired t-tests comparing pre to post scores revealed there was a significant difference for the typical class (*p*=0.005, *d*=1.1), the atypical class (*p*=0.03, *d*=0.7), but not for the walk condition (*p*=0.19). These results demonstrate that only the martial art classes were effective in improving the Interference scores.

## Discussion

To our knowledge, this is the first study to investigate the acute effects of martial art training on cognitive performance in healthy middle-aged men and women. Our results demonstrated that while all three exercise conditions improved attention and processing speed, only the martial art training improved the higher cognitive process of executive function.

Our results demonstrated that after the one-hour walk, the participants displayed improvements with large effect sizes in the Word and Color sections of the Stroop test, which are measures of attention and processing speed, but did not show improvements in executive function as measured by the Color-Word test and the Interference scores. Our results compare favorably to Barella et al. (2010) who also reported improvements in processing speed, but not in executive function using the Stroop test immediately following a twenty-minute walk in healthy older adults. Our results are also supported by Gothe et al. (2013) who demonstrated improvement in executive function following a yoga class, but not in the aerobic condition.

Chang et al. (2010) discussed the importance of investigating the cognitive effects of Tai Chi, a generic term representing the martial arts that originated from ancient China, as they are a form of mind-body exercise. Soo Bahk Do (SBD) is a Korean martial art that traces its roots back to the original Chinese martial arts. Both martial arts focus on the mind-body connection through concentration, mindfulness, energy management, proper movement patterns and breathing control. SBD has already been shown to improve physical fitness, the antioxidant system and arterial compliance of middle aged practitioners (Douris et al., 2004, 2009, 2013). The participants in this study, recreational middle aged SBD martial artists, all demonstrated significant improvements in attention, processing speed as well as executive function after the martial art classes. Both martial art classes displayed large effect size improvements for the Word test and a large and medium effect size improvement for the Color test after the typical and atypical class, respectively. The difference between the exercise conditions was their effect on executive function, which assesses and evaluates information so that an appropriate decision can be made. Executive function was measured by the Color-Word test and the Interference score which is also referred to as the Stroop effect. Both martial art classes improved in the Color-Word test and in the Interference score with the typical class causing a large effect size and the atypical class causing a medium effect size. Alves et al. (2014) reported improvements in the Color-Word section of the Stroop (executive function), in a high-intensity interval training condition compared to a light stretching condition. Martial art classes can be considered a type of interval training as high intensity exercises are interspersed with rest periods during the hour-long classes.

The results demonstrate that only the martial art classes were effective in improving the higher cognitive process of executive function possibly because increased cortical recruitment is necessary for the complex, coordinated motor demands of the martial art compared to the more repetitive actions of walking. The more repetitive actions of walking did not offer the same cortical stimulation and therefore only afforded improvements in attention and processing speed, but not executive function. Our secondary hypothesis, that the atypical martial arts class would have a greater effect on cognitive performance than the typical one was not supported.

The improvement in executive function seen after the martial art classes and not after walking may be attributed to our original hypotheses concerning the complexities of the movement patterns and associated cortical demands within each exercise condition. It has been reported that the regional cerebral blood flow increases along with the complexity of the movement patterns (Winstein et al., 1997). Therefore, it is possible that the martial art classes may have resulted in an increased cerebral blood flow to the areas of the brain responsible for executive function. According to Yanagisawa et al. (2010), functional neuroimaging showed that acute exercise activated the prefrontal cortex, resulting in improved executive function as measured by the Stroop test. Exercise has been shown to increase the middle cerebral artery blood flow which supplies the prefrontal cortex as well as the premotor, supplemental and motor cortex (Lyngeraa et al., 2013). Exercise may improve cognitive performance by increasing the cerebral blood flow which provides additional glucose, oxygen and energetic substances resulting in improved neurological functioning (Kashihara et al., 2009). It has been shown that complex motor tasks are more effective than simpler tasks in producing increased cortical excitability (Winstein et al., 1997; Yanagisawa et al., 2010; Carey et al., 2005). Therefore, it is possible that the martial art training may result in an increased cerebral blood flow to the areas of the brain responsible for executive function. The exercise–related cognitive benefits of complex movement tasks such as martial art training may be due to the increased neural stimulation imposed by the coordinative and complexity demands of the exercise.

There are limitations of this study that should be considered when interpreting its results. The intensity of a martial art class is difficult to quantify because of the interval nature of the class. Therefore, it was difficult to equalize the intensity of exercise between the martial art classes and the walking condition. However, we utilized the walking condition as the control taking into account that the research is overwhelming on its positive effect on cognitive performance (Barella et al., 2010; Alves et al., 2012; Tomporowski, 2003), and the purpose of this study was to investigate the effects of a more complex exercise condition on cognitive performance. Pesce (2012) stated the effects of complex movement type exercise as a mediator of cognitive performance had been previously under-investigated. However, undertaking of this study aimed to shed light on the quality of exercise experience and was not as concerned with the quantitative aspects of exercise as mediators of cognitive performance. Another limitation was that we utilized experienced martial artists as we required a specific skill set that they possessed and that they may not be representative of a typical middle-aged person, therefore, our results may not be extrapolated to the entire middle–aged population.

In conclusion, although the generalizabilty of the study is compromised by our specific sample, the obtained results demonstrate that continued research is warranted in the area of the acute effects on cognition of other types of physical activities that involve complex movement patterns in all age groups. Utilization of functional neuroimaging and measurement of the cerebral blow flow will assist in providing the physiological basis for changes in cognitive performance. We presented a prima fascie case for continued research on the role of challenging complex movement types of physical activity on acutely improving cognitive performance in the aging adult.

## Figures and Tables

**Table 1:** Physiological Characteristics of the Participants (n=10; six males and four females)

Age (years)	53.5 ± 8.0
Body height (cm)	170.1 ± 8.6
Body mass (kg)	75.9 ± 18.0

Values are given as mean ± standard deviation

**Table 2:** Stroop T-scores

Stroop T-Score	Word	Color	Color-Word	Interference
Pre Typical	49.4 ± 15.0	47.2 ± 10.8	53.1 ± 10.8	53.2 ± 10.3
Post Typical	57.6 ± 15.0†	54.2 ± 11.6†	59.6 ± 10.4†	58.9 ± 10.4†
Pre Walk	50.0 ± 10.9	47.1 ± 11.0	53.8 ± 12.3	53.7 ± 9.7
Post Walk	55.0 ± 10.4†	52.6 ± 11.6†	56.4 ± 11.5	55.4 ± 10.9
Pre Atypical	51.2 ± 8.5	48.3 ± 7.3	54.5 ± 9.8	53.6 ± 9.0
Post Atypical	58.6 ± 9.9†	53.4 ± 12.3*	57.9 ± 8.9*	57.0 ± 7.8*

Values are given as mean ± standard deviation

* Denotes a significant difference between pre and post test scores at p<0.05

† Denotes a significant difference between pre and post test scores at p<0.01
